# Recent Developments in Carbon Nanotube Membranes for Water Purification and Gas Separation

**DOI:** 10.3390/ma3010127

**Published:** 2010-01-04

**Authors:** Kallista Sears, Ludovic Dumée, Jürg Schütz, Mary She, Chi Huynh, Stephen Hawkins, Mikel Duke, Stephen Gray

**Affiliations:** 1 CSIRO Materials Science and Engineering, Bayview Ave, Clayton Vic 3168, Australia; E-Mails: ludovic.dumee@csiro.au (L.D.); jurg.schutz@csiro.au (J.S.); chi.huynh@csiro.au (C.H.); stephen.hawkins@csiro.au (S.H.); 2 Institute for Sustainability and Innovation, Victoria University, Werribee Campus, Hoppers Lane, Werribee PO Box 14428, Melbourne, Victoria, 8001, Australia; E-Mails: Mikel.Duke@vu.edu.au (M.D.); Stephen.Gray@vu.edu.au (S.G.); 3 Centre for Material and Fibre Innovation, Institute for Technology and Research Innovation, Deakin University, Geelong Vic 3217, Australia; E-Mail: mary.she@deakin.edu.au (M.S.)

**Keywords:** carbon nanotube, bucky-paper, membrane, filtration

## Abstract

Carbon nanotubes (CNTs) are nanoscale cylinders of graphene with exceptional properties such as high mechanical strength, high aspect ratio and large specific surface area. To exploit these properties for membranes, macroscopic structures need to be designed with controlled porosity and pore size. This manuscript reviews recent progress on two such structures: (i) CNT Bucky-papers, a non-woven, paper like structure of randomly entangled CNTs, and (ii) isoporous CNT membranes, where the hollow CNT interior acts as a membrane pore. The construction of these two types of membranes will be discussed, characterization and permeance results compared, and some promising applications presented.

## 1. Introduction

Many water and gas purification techniques, such as membrane distillation, reverse osmosis and CO_2_ removal from natural gas, are reliant on membranes. Consequently, the development of advanced membrane technologies with controlled and novel pore architectures is important for the achievement of more efficient and cost effective purification. Present polymeric membranes are well known to suffer from a trade off between selectivity and permeability, and in some cases are also susceptible to fouling or exhibit low chemical resistance.

**Figure 1:**
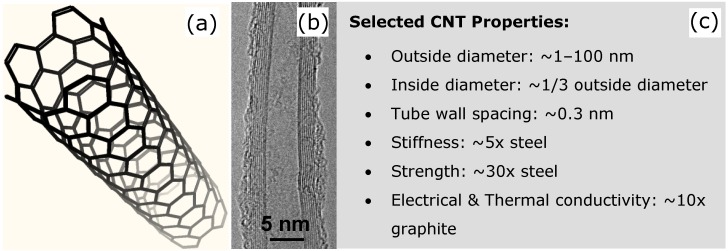
(a) Schematic of a CNT, (b) TEM image of a CNT showing a number of concentric graphitic walls, and (c) a list of selected CNT properties.

Membranes based on carbon nanotubes (CNTs) offer a possible route to overcome these shortcomings with a number of interesting structures emerging [1,2,3,4,5,6,7,8,9,10,11,12]. CNTs are nanoscale cylinders of rolled-up graphene (Figure 1) and can be capped at one or both ends with a half fullerene [13]. Single walled CNTs (SWNTs) have outer diameters in the range of 1–3 nm with inner diameters of 0.4–2.4 nm (Figure 1). Multi-walled CNTs (MWNTs) can have outer diameters ranging from ~2 nm (double walled nanotubes) up to ~100 nm with tens of walls. CNTs exhibit remarkable electrical and thermal conductivity, and are one of the strongest fibers known [14,15]. These properties, combined with their nanoscale dimensions, have led to their intense study for a wide range of applications [16]. However fabricating macroscopic structures which have controlled geometries, porosity and pore shape, is still challenging.

This paper reviews two types of CNT macroscopic structures under consideration for membrane applications: (i) Bucky-paper membranes and (ii) isoporous CNT membranes. These two types of CNT membranes are distinctively different in their structure and arrangement of the CNTs. In the case of a Bucky-paper, the CNTs are randomly arranged into a non-woven, paper-like, structure. This creates a highly porous 3D network with large specific surface area. In contrast, isoporous CNT membranes use aligned CNTs as cylindrical pores across an otherwise impermeable matrix material. These membranes therefore consist of nanoscale CNT pores with a narrowly defined diameter distribution. This paper reviews the various techniques used to fabricate these two types of CNT membranes and the associated challenges. It also discusses how the processing steps affect the final membrane structure, and presents some promising applications for water and/or gas separation.

## 2. Bucky-Paper Membranes

**Figure 2:**
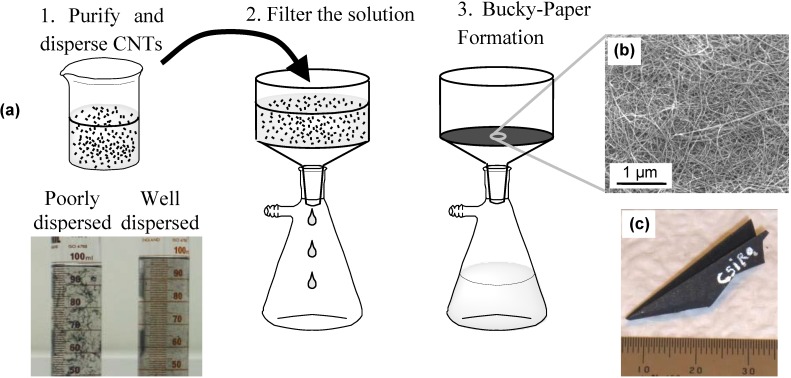
(a) Process for manufacturing Bucky-papers, (b) SEM image showing the Bucky-paper surface and (c) Bucky-paper origami aeroplane demonstrating their flexibility mechanical robustness.

Due to the simplicity of their preparation, Bucky-papers were one of the first macroscopic structures fabricated from CNTs and their mechanical, electrical and thermal properties have been extensively studied [12,17,18,19,20,21,22]. The term “Bucky-paper” is used to describe a mat of randomly entangled CNTs prepared by filtration (Figure 2) [21,23] or alternative papermaking processes. CNTs are known to have a strong tendency to aggregate due to van der Waals interactions, and it is these van der Waals interactions which also hold the CNTs together into a cohesive Bucky-paper. Consequently Bucky-papers can be highly flexible and mechanically robust as demonstrated by the origami plane in Figure 2c. Longer, narrower (fewer walled) and more pure nanotubes typically lead to stronger Bucky-papers with higher tensile strengths [18,21,24]. With increasing MWNT diameter, the attractive van der Waals forces between CNTs become less effective, leading to Bucky-papers with lower tensile strength and poor cohesiveness. This can be improved to some extent through functionalization of MWNTs or the addition of polymers [18].

### 2.1. Bucky-Paper Processing

Bucky-papers are typically formed by first purifying the CNTs and then dispersing them in a suitable solvent. Once a well dispersed solution is achieved, it is filtered through a porous support which captures the CNTs to form an optically opaque CNT Bucky-paper (Figure 2). If the Bucky-paper is thick enough it can be peeled off the support filter intact.

**Figure 3:**
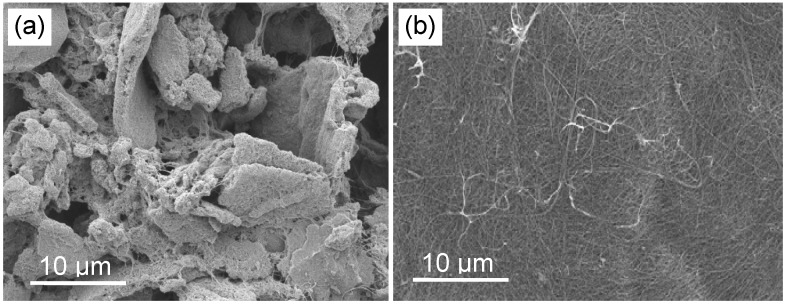
SEM image showing the surface of a Bucky-paper formed from (a) poorly dispersed single walled carbon nanotubes and (b) well dispersed CNTs (2 keV, 9 mm working distance).

As prepared CNTs are highly entangled and typically contaminated with impurities. These impurities include the metal catalyst particles, such as Fe, Co and Ni needed for CNT growth, as well as other carbonaceous by-products including amorphous carbon, fullerenes, and graphitic nano-particles. The purification and dispersion of CNTs is therefore a critical step in Bucky-paper processing that can affect both the Bucky-paper structure and properties [25,26,27]. This point is illustrated by Figure 3 which clearly shows the change in Bucky-paper morphology due to differences in the initial CNT dispersion quality.

Purification inevitable employs some form of oxidative treatment in combination with physical processes such as filtration and centrifugation [28]. Nitric acid (HNO_3_) or heating in an oxidative environment (e.g., air) is commonly used to remove carbonaceous impurities, which are oxidised at a faster rate than CNTs due to their less perfect graphitic structure. This treatment is often preceded and/or followed by another acid treatment, such as hydrochloric acid (HCl), to remove any metal impurities [24,29,30,31]. However these purification treatments can also damage and shorten the CNTs, as well as functionalize them with carboxyl and hydroxyl groups rendering them hydrophilic [32,33]. This can advantageous for CNT dispersion into polar solvents such as water. Improved Bucky-paper strength has also been reported following acid treatment and CNT functionalization under controlled conditions [18]. However these purification steps also alter the natural CNT properties, which may not be desirable for the final application.

CNT dispersion typically involves one or a combination of the following the following approaches [34,35]:

(i) covalent functionalization of the CNT surface to improve their chemical compatibility with the dispersing medium [36,37].
(ii) the use of a third component such as a surfactant [34,35,38,39,40], polymer [41] or biomolecules (such as DNA [42]).
(iii) mechanical treatments such as ultrasonication and shear mixing.

Again, the dispersion steps need to be carefully chosen to suit the type of CNTs and the final application so that the desired CNT properties are not adversely affected [24]. Further details on purification and dispersion techniques can be found in a number of articles and reviews [18,24,34,37,43,44].

### 2.2. Bucky-Paper Structure and Properties

**Figure 4:**
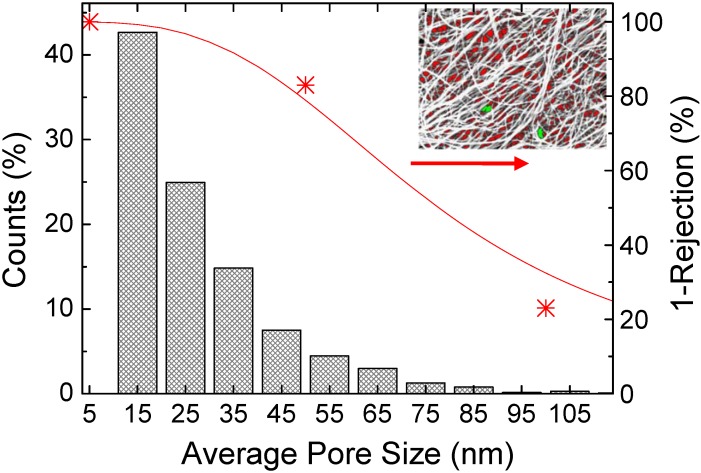
(Histogram - left axis): Average pore size distribution determined by SEM imaging of a Bucky-paper. (Red stars – right axis): Particle rejection tests with polystyrene spheres.

As illustrated in Figure 2b, Bucky-papers tend to form a highly porous network of randomly orientated CNTs, although the CNTs predominately lie in a plane parallel to the Bucky-paper surface. The high porosity and random CNT arrangement are particularly evident in movie S1 (supplementary information) which shows a series of TEM images taken with increasing sample tilt from 30 to 150°. Although Bucky-paper membranes do not consist of well defined pores of a single characteristic shape and size, SEM imaging of the surface followed by image analysis, is useful for calculating an “apparent surface” pore size as shown by the histogram and inset of Figure 4 [12,45,46]. The analysis in Figure 4 is for a Bucky-paper fabricated from MWNTs grown by Chemical Vapor Deposition (CVD) that have an average outer diameter and length of 9 nm and ~300 µm, respectively (see Table 1–fine CNTs). The CNTs were dispersed in analytical grade isopropanol by repeated sonication and stirring, and then immediately filtered though a poly(ether-sulfone) (PES) support of 0.22 µm pore size to form the Bucky-paper. No acid treatment or purification steps were used in order to preserve the CNT’s inherent hydrophobicity (see section 2.3.1). The resulting structure was nevertheless of high purity (>95% CNT) due to careful choice of the CVD growth technique and parameters. While the average pore size is small, ~25 nm, the pore size distribution is quite broad with a standard deviation of ~14 nm. This is consistent with pore size distributions reported by other groups for similar MWNT Bucky-papers [47], where the average pore size was 29–39 nm with standard deviations of 10–20 nm [48,49]. Figure 4 (stars/right axis) also shows results from particle (polystyrene) rejection tests for the same Bucky-paper. These are in reasonable agreement with the pore size distribution determined from SEM with a rejection of 80% for 100 nm diameter spheres and 98% for 500 nm diameter spheres (not shown).

**Figure 5:**
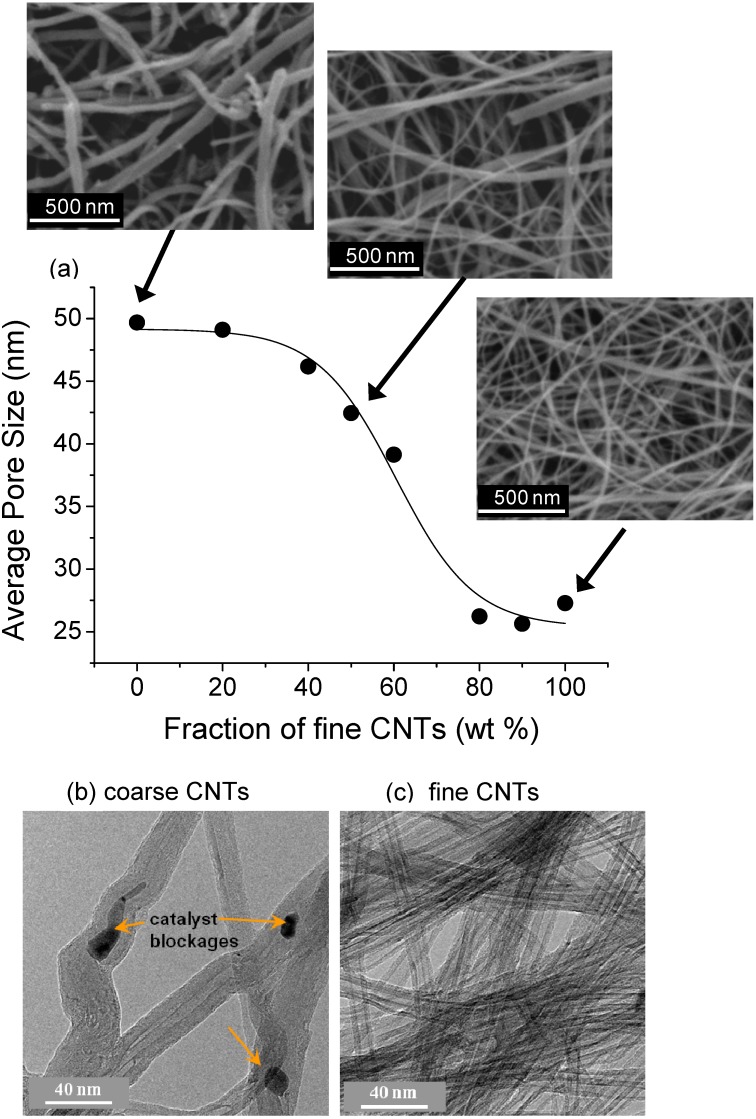
(a) Dependence of the Bucky-paper pore size on the ratio of “fine to coarse” carbon nanotubes. The SEM images show the Bucky-paper surface for three different ratios as indicated. (b) and (c) are TEM images (200 kV) of the coarse and fine nanotubes, respectively. The orange arrows indicate catalyst particles within the CNTs. The CNT properties are also summarized in Table 1.

**Figure 6:**
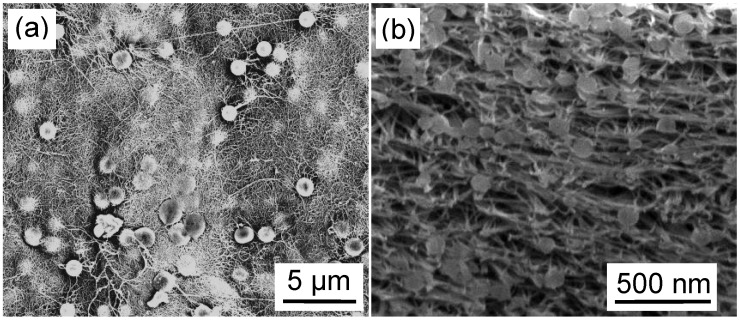
SEM images showing (a) the surface and (b) a cross section (52° sample tilt) of a Bucky-paper formed from a mixed dispersion of PS beads and multi-walled carbon nanotubes (5 kV, 5 mm working distance). The PS beads in (a) and (b) had 1 μm and 100 nm diameters, respectively. The cross-section was formed by milling with a Gallium Focused Ion Beam.

The Bucky-paper pore size is also highly dependent on the type of CNTs used. Figure 5 illustrates how this can be used to tune the average pore size by mixing two types of CNTs in different ratios. The two types of CNTs are referred to as fine (~9 nm outer diameter) and coarse (~37 nm outer diameter) with reference to their outer diameter and other structural properties (Table 1 and Figure 5b-c). Bucky-papers formed solely from fine or coarse CNTs had an average surface apparent pore size of ~25 and 49 nm, respectively, while intermediate pore sizes were obtained by using mixtures of the two (Figure 5). Several groups have shown other ways to control porosity and pore size. Kukovecz *et al*. changed the Bucky-paper pore size through the CNT length, which was varied from 2 μm down to 230 nm through a ball milling treatment [48]. Das *et al*. controlled the porosity by dispersing polymer beads together with the CNTs to form a Poly(Styrene) (PS)/CNT Bucky-paper composite [50]. The polymer beads were subsequently dissolved creating voids in the Bucky-paper. Figure 6 shows surface and cross-sectional SEM images of a similar composite structure formed by our group using polystyrene beads from Sigma Aldrich (L1528).

**Figure 7:**
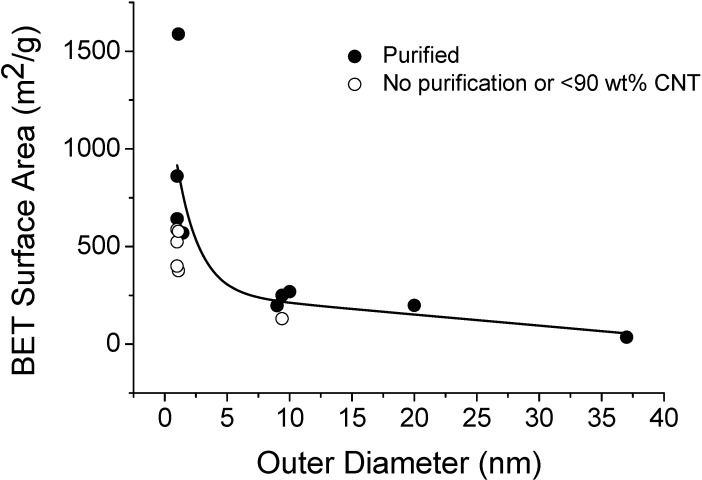
Dependence of carbon nanotube specific surface area on nanotube outer diameter. The data was taken from the literature and supplemented with our own measurements (see Table S1, supplementary materials, for further details and references for the data). The open circles represent results for nanotubes reported to have high impurity content.

Bucky-papers offer incredible porosity and specific surface area. Helium pycnometer measurements made on Bucky-papers fabricated from the fine and coarse CNTs discussed earlier, indicated porosities of 91% and 87%, respectively. Furthermore Cinke *et al*. reported a specific surface area as high as 1587 m^2^/g for Bucky-papers formed from SWNTs [29]. They attributed this high surface area to their two step purification process which ensures that the CNTs are de-bundled and highly pure. Figure 7 compares values of specific surface area reported in the literature by plotting them as a function of the CNT outer diameter. As expected a monotonic decrease in the specific surface area is observed with increasing diameter. Since nitrogen cannot penetrate into the space between concentric graphene walls of MWNTs, the specific surface area to CNT mass decreases with increasing CNT outer diameter. The data points represented by open circles in Figure 7 are from CNT samples for which a high impurity content was reported. Judging from the significantly lower surface areas that have been measured for these samples, it seems plausible that the specific surface area is higher once the impurities have been removed.

**Table 1:** Properties of the coarse and fine carbon nanotubes (CNTs) grown by CVD.

CNT Type	Coarse	Fine
**Inner diameter (nm)**	10 ± 5.5	4.5 ± 1
**Outer diameter (nm)**	37 ± 16	9 ± 1.5
**# walls**	37 ± 21	6 ± 2
**Length (µm)**	200–400	200–400
**Impurity content**	<10 wt %	<5 wt %

**Figure 8:**
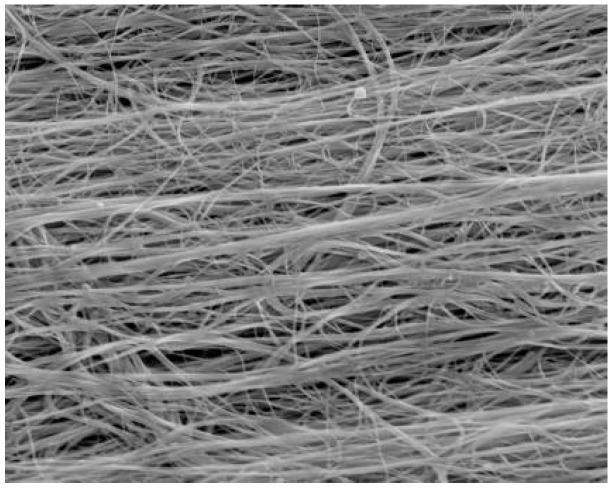
SEM image showing the surface of an aligned carbon nanotube Bucky-paper (5 kV, 5 mm working distance).

Several authors have investigated methods to introduce CNT alignment in Bucky-papers (Figure 8) [51,52]. Their results indicated enhanced conductivity along the alignment direction and that the porosity and pore structure are also likely to be affected [53,54].

### 2.3. Bucky-Papers for Water Purification and Filtration

#### 2.3.1. Membrane Distillation

**Figure 9:**
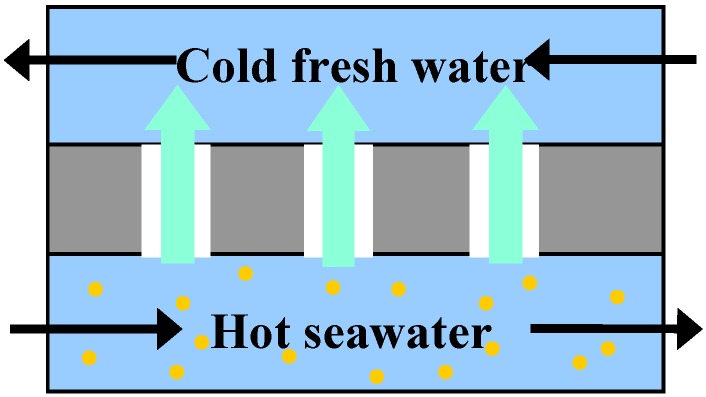
Principle behind direct contact membrane distillation.

**Figure 10:**
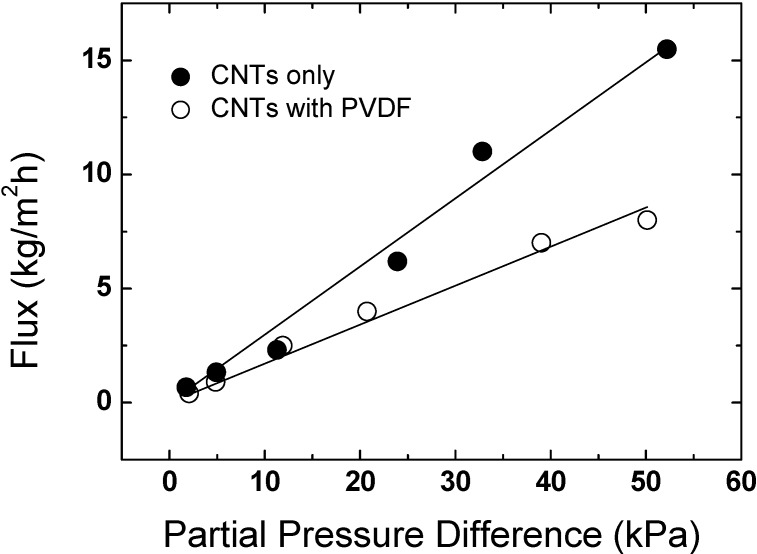
Dependence of water vapor flux on the partial pressure difference across a Bucky-paper membrane in a direct contact membrane distillation setup [stream flux 300 mL/min; salt concentration ~35 g/L; T_cold_ ~5 ºC, T_hot_ varied from 25 to 95 ºC].

In terms of the application of Bucky-papers, the authors’ work concentrates on their use for water purification by a process called direct contact membrane distillation. This technique is an alternative to reverse osmosis and other desalination techniques, particularly when the concentration of solutes is high [55]. As illustrated in Figure 9, the Bucky-paper is used as a highly hydrophobic membrane to separate a feed of hot sea or brackish water from a permeate of cold fresh water. While liquid cannot cross the air gap formed by the membrane, water vapor is able to pass through the pores from the hot feed to the cold permeate driven by the difference in partial vapor pressure. This vapor then condenses on the permeate side creating fresh water. The inherent hydrophobicity of the nanotubes (D.I. water contact angle ~113°) and high Bucky-paper porosity (~90%) lend them to this application and we have demonstrated water vapor permeabilities of up to 3.3 × 10^-12^ kg/m sPa on a small scale rig [12,45]. However, cracking of the Bucky-papers with time is a problem as salt water can penetrate into the relatively large cracks and breach the Bucky-paper membrane. This leads to a gradual reduction in permeate quality over time [12]. Figure 10 demonstrates how the vapor flux increases with the difference in partial vapor pressure across the membrane. The two curves in Figure 10 represent results from a pure Bucky-paper (solid circles) and from a composite Bucky-paper (open circles). The composite Bucky-paper was created by vacuum filtering a solution of PVDF through the Bucky-paper structure. The PVDF forms a thin coating on the CNTs which decreases the tendency for the Bucky-paper to crack and hence improves its operational lifespan. This however comes at the expense of a lower porosity and hence reduced flux and permeability [45].

#### 2.3.2. Other Applications

Bucky-papers have also been considered for a number of other applications related to filtration and water purification. Several groups have demonstrated desalination of low salinity (<~5000 mg/L) water using Bucky-paper like structures in a capacitive de-ionization setup [56,57,58,59,60]. This application takes advantage of the electrical conductivity and high porosity offered by Bucky-papers. The setup comprises two electrodes arranged to form a parallel plate capacitor across which a voltage is applied to absorb salt ions of opposite polarity from a stream of salty water. The salt is then released as a concentrated brine when the applied potential is reversed. Wang *et al*. demonstrated an electrosorption capacity of ~57 μmol/g, which was similar to a carbon aerogel electrode despite its lower surface area and is attributed to the more optimal pore size distribution of the Bucky-paper [58].

Bucky-papers have also been used as fine filters. Viswanathan *et al*. reported that a 2 µm thick CNT Bucky-Paper film supported on a cellulose acetate disc was capable of filtering fine particles of 100–500 nm diameter to a level that exceeded the standards set out for HEPA filters [61]. They also suggest that these Bucky-Papers could be used to filter powdered organic dyes and condensed lead fumes.

Antimicrobial properties (in the absence of UV/vis irradiation) and the efficient removal of bacterial from contaminated waters have also been demonstrated [4,62,63]. Brady-Esétvez *et al*. demonstrated that a SWNT Bucky-paper was effective in completely retaining E. coli cells (2 μm size) due to size exclusion and also exhibited exceptionally high removal of the model virus MS2 bacteriophage (27 nm diameter) due to depth filtration. Furthermore the SWNT Bucky-paper promoted the inactivation of E. coli cells which was attributed to cell membrane damage on direct contact with SWNT aggregates [62]. Although it is also worth mentioning that other groups have reported fibroblast cell attachment and proliferation on CNT based scaffolds and Bucky-papers [64,65,66,67].

## 3. Isoporous Carbon Nanotube Membranes

**Figure 11:**
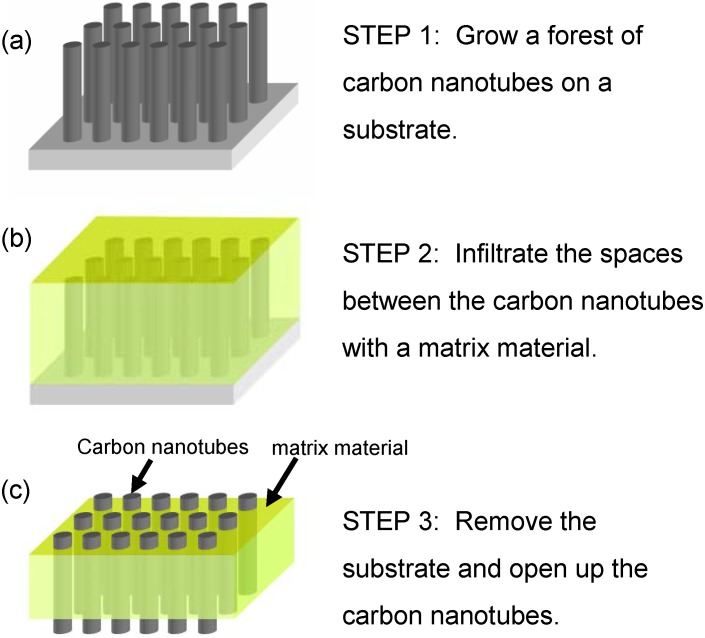
Schematic showing the general approach used to construct isoporous CNT membranes.

This structure is different from a Bucky-paper in that it uses the CNTs as cylindrical pores across an otherwise impermeable thin film (Figure 11c). This results in a membrane with well controlled nanoporosity with the only route for flow through the hollow CNT interior. These structures are promising for high permeability, high selectivity membranes due to the small CNT diameter (as small as 0.7 nm) and predictions of rapid flux through their hollow interior [68,69,70,71,72]. Molecular dynamic simulations have also shown that CNT membranes, in theory, can be used for desalination via reverse osmosis [73].

The predicted rapid flux through CNTs is attributed to two factors. First and foremost is the CNT’s smooth, frictionless interior. This is predicted to result in specular, instead of diffusive, collisions between molecules and the CNT wall, leading to enhanced flow for (i) gases in the Knudsen regime [4,69,70] and (ii) pressure driven liquid flow through a pipe (classically described by the Hagen- Poiseuille law) [74]. Secondly, for CNTs with diameters less than ~2 nm, molecular ordering and single file diffusion have been predicted to lead to the concerted movement of molecules [68,71,73,75,76,77,78]. In particular, Hummer *et al*. predicted ballistic motion of water chains through the CNT interior due to strong hydrogen bonding between water molecules and minimal interaction with the CNT wall [68].

While it is intuitively unfavorable for a polar molecule, such as water, to enter the non-polar interior of a CNT, experimental evidence seems to indicate otherwise [1,2,3,11,79,80,81,82,83,84]. One of the first demonstrations of liquid flow through a CNT was by Sun *et al*. who embedded an individual MWNT (inner diameter 150 nm) into an epofix epoxy resin followed by microtoming to form thin membrane slices [80]. However it was the work of two separate groups, Hinds (Majumder) *et al*. and Holt *et al*., that caught the interest of the scientific community [1,2,3]. Both groups independently fabricated membranes with a high density of aligned CNT pores and demonstrated fluid flow 2–3 orders of magnitude greater than that predicted by conventional fluid flow theory, although their results have been questioned by some [74]. Both of these groups have also reported functionalization of the CNT tips to gate fluid flow through the CNT pores or enhance their selectivity [85,86,87,88,89]. Since these findings, a number of groups have reported on the construction and permeance of isoporous CNT membranes (Table S2). In the following section the different approaches to membrane construction will be reviewed. While Holt *et al*. and Hinds *et al*. are still the only two groups to have reported water flow through the interior of CNT pores, a number of groups have reported permeance for gases. Consequently gas permeance is a useful parameter by which to compare the various approaches to membrane construction and is discussed in section 3.2.

### 3.1. Membrane Construction

**Figure 12:**
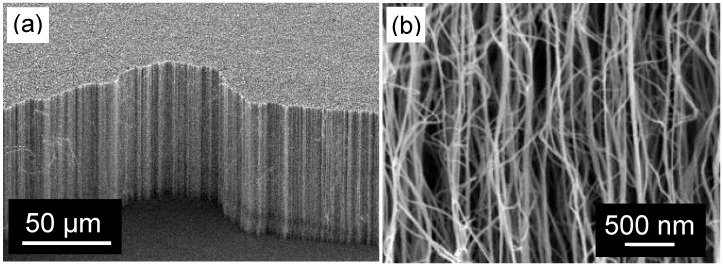
SEM images of a CNT forest grown by chemical vapor deposition on a silicon substrate: (a) low magnification (~35° sample tilt) and (b) higher magnification (2 kV, 9 mm working distance).

For membrane construction, most groups use variations on the general approach outlined in Figure 11 [1,2]. Construction typically begins with a forest of aligned CNTs grown by Chemical Vapor Deposition (CVD) on either a silicon or quartz substrate (Figure 12). The growth parameters need to be carefully chosen so that the CNTs are free from structural blockages such as catalyst particles or bamboo type structures, which could prevent flow through the CNT interior [90]. Once a CNT forest is grown, the spaces between CNTs are infiltrated with an impermeable material to form a continuous matrix. Finally, the excess matrix material and substrate are removed, opening up the CNT ends.

**Figure 13:**
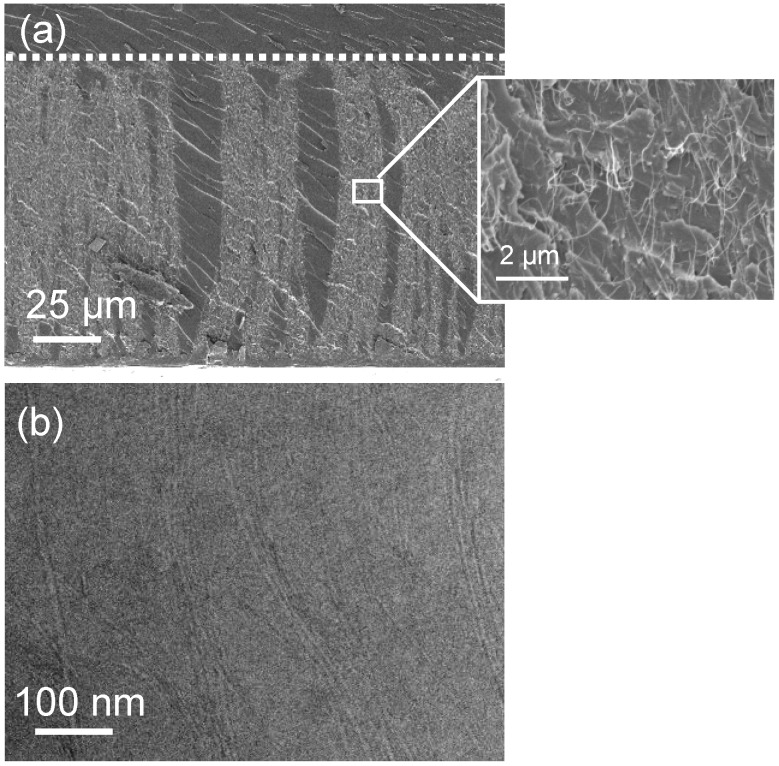
Images of a CNT forest after infiltrating with epoxy (a) SEM image of a liquid nitrogen fractured cross-section. The CNTs are compacted slightly into columns (bright regions) due to surface tension effects, leaving CNT free regions (dark) in between. (b) TEM image (sample prepared by focus ion beam milling). The contrast between the CNTs and epoxy is low due to their similar carbon based composition.

**Figure 14:**
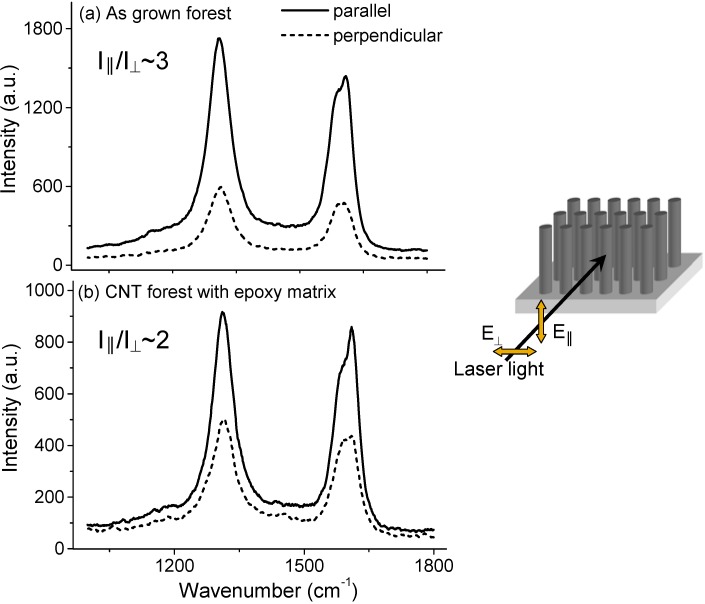
Raman spectra taken from (a) an as grown CNT forest and (b) a forest after infiltrating with epoxy. Both the D (1,310 cm^-1^) and G (1,590 cm^-1^) bands are present. The Raman signal intensity is sensitive to the CNT alignment and is strongest when the incident polarization is parallel to the CNT axis. It can therefore give an indication of the CNT alignment. A 783 nm laser with incident power of 2 × 10^4^ W/cm^2^ was used to avoid luminescence from the epoxy resin (see supplementary material).

In our method we form a matrix by infiltrating the CNT forest with a two part, low viscosity epoxy followed by curing at 80–120 °C [91]. Most importantly, the matrix needs to be void free, while at the same time preserving the CNT alignment. Figure 13 shows SEM and TEM images of one of our CNT forests after embedding with epoxy. Clearly, the epoxy conforms well to the CNTs without any obvious cracks or voids. Due to surface tension effects during infiltration, the CNTs are densified into columns (bright contrast) creating CNT free regions in between (Figure 13a). However the forest height before and after infiltration remains the same indicating that the CNT alignment is largely maintained. To further investigate the degree of CNT alignment, Raman spectra were measured for an as grown forest and one which had been epoxy infiltrated (Figure 14). The Raman intensity is sensitive to the CNT alignment and is strongest for incident light polarized parallel to the CNT axis [92,93,94]. A qualitative measure of the CNT alignment is therefore possible by measuring the intensity ratio, I_||_/I_⊥,_, for parallel and perpendicularly polarized light. Values of 3 and 2 were determined for the as grown and epoxy infiltrated forests respectively, indicating some loss of alignment. Vapor phase infiltration, such as that used by Holt *et al*., may better preserve the CNT alignment [1].

**Figure 15:**
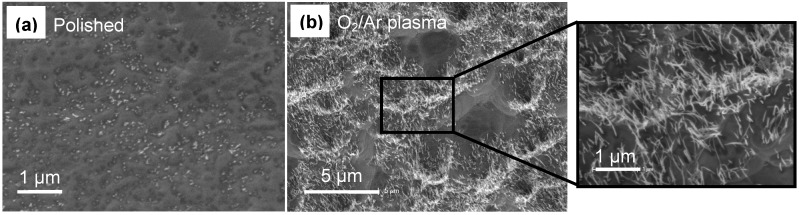
SEM images showing the CNT membrane surface after (a) polishing and (b) a 4 hour plasma treatment with a mixture of 30% O_2_ in Argon. The high frequency plasma treatments were performed at a pressure of 0.6 mbar and power of 80W in a Pico PC system from Diener Electronics.

To remove the excess matrix material and open up the CNTs a number of treatments have been employed, such as polishing, acid treatment and oxygen (H_2_O or O_2_) based high frequency plasma treatments (at 13.56 MHz) [1,2,10]. Figure 15 shows SEM images of our epoxy infiltrated forest after first polishing with diamond paste and then plasma etching with a 30% O_2_/Ar mixture. Few CNTs (bright contrast) are visible after polishing, while many more are exposed by the plasma treatment (Figure 15b). This is reflected in the air permeance which increased by an order of magnitude from ~1 × 10^-10^ to ~1 × 10^-9^ moles/m^2^/s/Pa after plasma treatment. However this is still an order of magnitude lower than that predicted by Knudsen diffusion for a CNT density of 5 × 10^10^ cm^-2^, based on the as grown CNT forest, indicating that the majority of CNTs are not yet contributing to permeance and further treatments are necessary.

A number of groups have taken slightly different approaches to that outlined in Figure 11. Mi *et al*. grew the initial CNT forest directly onto a macroporous alumina substrate [10]. The alumina substrate acts as a support for the final CNT membrane and avoids the etching or polishing step required for non-porous silicon or quartz substrates. However it comes at the expense of a reduced CNT density which is reflected in the gas permeance discussed in Section 3.2.

Even at a typical forest density of 10^11^ cm^-2^ and a CNT inner diameter of 5 nm, the total CNT areal coverage and hence porosity is less than 2%. To improve the available area for permeation, Yu *et al*. fabricated a dense block of aligned CNTs by shrinking an as grown CNT forest [95]. To shrink the forest they first detached it from the substrate by a water etching step and then collapsed it into a single block using the capillary forces generated by solvent evaporation [96,97]. Yu *et al*. did not apply a matrix material to the condensed forest so that fluid flow is possible through both the CNT interior and gaps between CNTs, which they estimated were less than 3 nm [95].

Kim *et al*. have reported on a scalable method of fabricating membranes which involves first dispersing amine-functionalized CNTs in tetrahydrofuran (THF) and filtering this solution through a porous PTFE substrate [6]. The CNTs appear to spontaneously align themselves perpendicular to the porous PTFE substrate in the draft of the fluid. These CNTs are then embedded in a polysulfone layer. Surprisingly, the polysulfone layer does not seem to block all of the CNTs as they report gas permeance without applying further surface treatments.

Finally, an approach used by several other groups is to grow CNTs within the pores of an anodized alumina template. This leads to forests of vertically aligned, straight CNTs within an alumina matrix. However it appears that these CNTs are only semi-graphitic [11,79,98,99]. As such they do not possess the inherent smoothness and hydrophobicity of a purely graphitic CNT and may not exhibit the same fluid flow properties.

### 3.2. Gas Permeance

**Figure 16:**
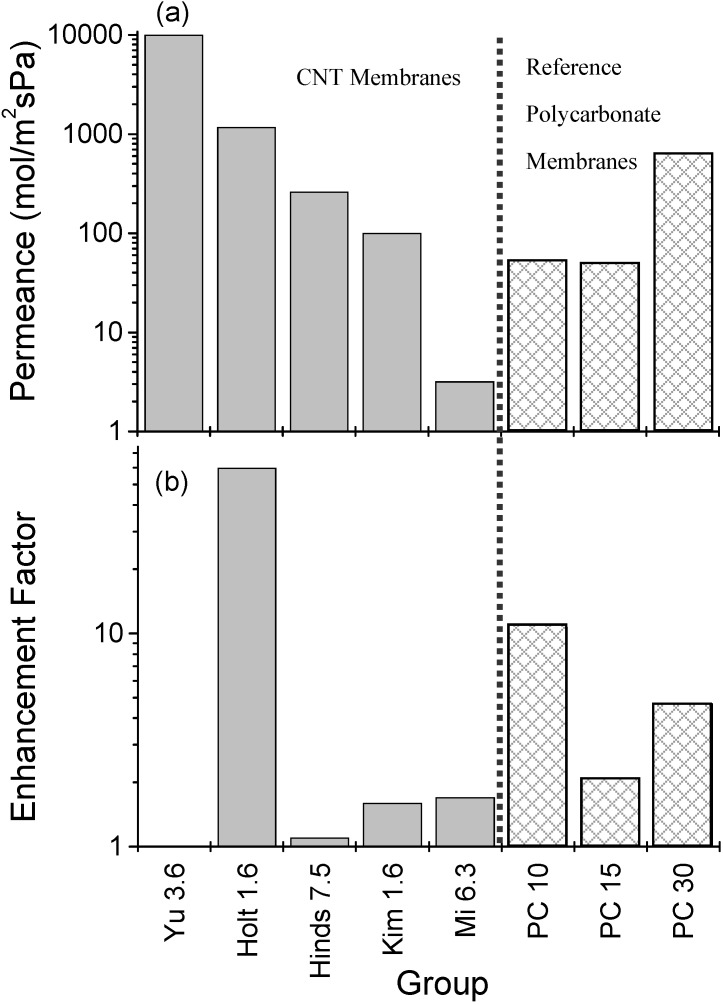
Summary of gas permeance values reported in the literature for isoporous CNT membranes: (a) Permeance and (b) Enhancement Factor which is defined as the measured permeance divided by the permeance predicted assuming Knudsen flow. An enhancement value is not given for Yu *et al*. as gas flow is through both the CNT hollow interior and gaps between CNTs. The last three cross-hatched bars are for polycarbonate track etched membranes. PC10 and PC30 were measured by the authors, while PC15 is taken from [1]. The pore diameter (in nm) is given after the name of each group.

Gas permeance is a useful method for evaluating membrane performance and is compared in Table S2 and Figure 16a for the different isoporous CNT membranes discussed above. Here we define permeance as the flux through the membrane divided by the membrane area and the differential pressure. The comparison in Figure 16a suggests that membrane permeance is most affected by the CNT pore density. For example, the highest permeance in Figure 16a is for the membrane reported by Yu *et al*. which was formed by densifying an as grown forest [95]. In contrast the membrane structure reported by Mi *et al*., which was based on a low density CNT forest, has a permeance three orders of magnitude lower [10].

Figure 16 also shows permeance results for three track etched polycarbonate (PC) membranes with 10, 15 and 30 nm diameter pores. The 10 and 30 nm membranes were measured by the authors while the value for the 15 nm PC membrane was taken from Holt *et al*. [1]. The PC membranes consist of well defined cylindrical pores and therefore offer an ideal benchmark for the CNT membranes. Despite the smaller CNT diameter, the CNT membranes are able to achieve a similar if not better permeance than the PC membranes. For example, the gas permeance for CNT membranes reported by Yu, Holt and Kim are ~200, ~20 and ~2 times that of a commercial 10 nm PC membrane, respectively. This is possible, in part, due to a higher CNT pore density compared to PC. As discussed below in relation to the enhancement factor, the atomically smooth and hydrophobic surface of CNTs may also contribute to their high gas permeance, especially for small diameter CNTs (<~2 nm).

Figure 16b compares the enhancement factor for the same CNT membranes and enables a more direct comparison between them as it takes into account differences in membrane thickness, CNT diameter and CNT density. The enhancement factor is defined here as the experimental permeance (plotted in Figure 16a) divided by the permeance predicted from Knudsen theory. Knudsen diffusion applies when the mean free path divided by pore radius is greater than one, which is the case for all of the membranes reviewed here (see Table S2). In the Knudsen regime, there are more collisions with the CNT wall than with other molecules and the permeance, *f_k_* [moles/m^2^/s/Pa] is given by:

(1) $$f_{K}=\frac{\varphi }{3}\sqrt{\frac{8}{\pi RTM}}\frac{1}{\tau \;L}\rho_{pores}\times A_{pore}$$

where *φ* [m] is the CNT diameter, *R* [J moles^-1^ K^-1^] is the universal gas constant, *T* [K] is the temperature, *M* [kg/mole] is the gas molecular weight, *τ* is the pore tortuosity, *L* [m] is the membrane thickness, *ρ_pores_* [m^-2^] is the density of pores, and A*_pore_* [m^2^] is the inner area of each CNT pore and is equal to *π φ^2^/4*. The biggest uncertainty in determining the enhancement factor lies in an accurate knowledge of the density of CNTs contributing the membrane’s permeance. Typically SEM or TEM imaging has been used to estimate an upper value [1,6,10].

The enhancement factor lies between 1–2 for most of the CNT membranes (Figure 16b), which is in reasonable agreement with Knudsen diffusion considering the uncertainty linked to many of the input values, in particular the CNT density. The exception to this is Holt *et al*. who report a phenomenal enhancement factor of ~60 [1]. This enhancement over Knudsen diffusion in likely due their sophisticated fabrication route, which may ensure a greater percentage of open CNTs, in combination with the small CNT diameter used, 1.6 nm. For small CNT diameters, enhanced flow rates are predicted due to nano-scale confinement and the smooth, hydrophobic CNT interior [68,72]. Interestingly, the PC membranes show enhancement factors between 2–10. This may be due to a non-uniform pore diameter throughout the membrane thickness. Alternatively, other non-Knudsen transport mechanisms such as viscous flow may also be contributing to their permeance.

In terms of gas separation, most reported studies found that the single-component selectivity exhibited an inverse-square-root scaling with molecular mass, characteristic of Knudsen diffusion [1,6,10,95]. Holt *et al*. found that hydrocarbons were an exception to this and exhibited higher selectivities [1]. This was attributed to the preferential interaction of hydrocarbons with the CNT internal walls and possibly surface diffusion. Hence it may be possible to separate mixtures such as CO_2_/CH_4_ through this mechanism.

## 4. Conclusions

In summary, this paper has reviewed the fabrication and application of two types of CNT based membranes (i) Bucky-papers and (ii) isoporous CNT membranes. Both of these membranes have distinctively different structures and porosity. Bucky-paper membranes are comprised of randomly entangled CNTs that are fabricated by a relatively simple process involving vacuum filtration. The Bucky-paper properties depend on the type of CNTs used and their pre-treatment (purification and dispersion). They typically offer a highly porous structure with large specific surface area. As such they are of interest for applications such as direct contact membrane distillation, capacitive de-ionization, and filtration of particles including bacteria and viruses. In contrast, isoporous CNT membranes use the CNTs as pores across an otherwise impermeable matrix material. A handful of groups have published different approaches to isoporous CNT membrane construction with promising permeance results. Despite the smaller CNT diameter, gas permeances equal to or higher than that of commercial polycarbonate membranes with cylindrical, 10 nm diameter pores, have been reported. This is made possible, in part, by a higher CNT pore density compared to polycarbonate membranes. However, as demonstrated by Holt *et al*., flow enhancement due to the atomically smooth and hydrophobic CNT surface may also play a large role for CNT pore diameters less than ~2 nm. Several groups have also demonstrated fast liquid flow through the CNT interior, 2–3 orders of magnitude greater than that predicted by conventional theory, and seem to confirm theoretical predictions. These isoporous membranes are therefore of great interest for nanofiltration membranes with both high flux and selectivity. One of the major challenges lies in fabricating large scale isoporous CNT membranes, while still maintaining their structural integrity.

## Supplementary Materials

Movie S1 showing TEM images of a Bucky-paper membrane with sample tilts of 30–150°.

Experimental Details, Table S1 and Table S2.

Supplementary materials can be downloaded at http://www.mdpi.com/1996-1944/3/1/127/s1.

## References

[1] Holt J.K., Park H.G., Wang Y., Staderman M., Artyukhin A.B., Grigoropoulos C.P., Noy A., Bakajin O. (2006). Fast Mass Transport Through Sub-2-Nanometer Carbon Nanotubes. Science.

[2] Hinds B.J., Chopra N., Rantell T., Andrews R., Gavalas V., Bachas L.G. (2004). Aligned Multiwalled Carbon Nanotube Membranes. Science.

[3] Majumder M., Chopra N., Andrews R., Hinds B.J. (2005). Enhanced Flow in Carbon Nanotubes. Nature.

[4] Srivastava A., Srivastava O.N., Talapatra S., Vajtai R., Ajayan P.M. (2004). Carbon Nanotube Filters. Nature.

[5] Li X., Zhu G., Dordick J.S., Ajayan P.M. (2007). Compression-Modulated Tunable-Pore Carbon-Nanotube Membrane Filters. Small.

[6] Kim S., Jinschek J.R., Chen H., Sholl D.S., Marand E. (2007). Scalable Fabrication of Carbon Nanotube/Polymer Nanocomposite Membranes for High Flux Gas Transport. Nano Lett..

[7] Kim S., Pechar T.W., Marand E. (2006). Poly(imide siloxane) and Carbon Nanotube Mixed Matrix Membranes for Gas Separation. Desalination.

[8] Peng F., Pan F., Sun H., Lu L., Jiang Z. (2007). Novel Nanocomposite Pervaporation Membranes composed of Poly(vinyl alcohol) and Chitosan-Wrapped Carbon Nanotubes. J. Membr. Sci..

[9] Peng F., Hu C., Jiang Z. (2007). Novel Poly(vinyl alcohol)/Carbon Nanotube Hybrid Membranes for Pervaporation Separation of Benzene/Cyclohexane Mixtures. J. Membr. Sci..

[10] Mi W., Lin Y. S., Li Y. (2007). Vertically Aligned Carbon Nanotube Membranes on Macroporous Alumina Supports. J. Membr. Sci..

[11] Whitby M., Cagnon L., Thanou M., Quirke N. (2008). Enhanced Fluid Flow through Nanoscale Carbon Pipes. Nano Lett..

[12] Dumée L.F., Sears K., Schütz J., Finn N., Huynh C., Hawkins S., Duke M., Gray S. (2009). Characterisation and Evaluation of Carbon Nanotube Bucky-paper Membranes for Direct Contact Membrane Distillation. J. Membr. Sci..

[13] Iijima S. (1991). Helical Microtubules of Graphitic Carbon. Nature.

[14] O'Connell M.J. (2006). Carbon Nanotubes: Properties and Applications.

[15] Dresselhaus M.S., Dresselhaus G., Avouris Ph. (2001). Carbon Nanotubes: Synthesis, Structure, Properties, and Applications.

[16] Baughman R.H., Zakhidov A.A., De Heer W.A. (2002). Carbon Nanotubes-the Route toward Applications. Science.

[17] Zhang X., Sreekumar T.V., Liu T., Kumar S. (2004). Properties and Structure of Nitric Acid Oxidized Single Walled Carbon Nanotube Films. J. Phys. Chem. B.

[18] Xu G., Zhang Q., Zhou W., Huang J., Wei F. (2008). The Feasibility of Producing MWCNT Paper and Strong MWCNT Film from VACNT Array. Appl. Phys. A: Mater. Sci. Process..

[19] Bandow S., Rao A.M., Williams K.A., Thess A., Smalley R.E., Eklund P.C. (1997). Purification of Single-Wall Carbon Nanotubes by Microfiltration. J. Phys. Chem. B.

[20] Baughman R.H., Cui C., Zakhidov A.A., Iqbal Z., Barisci J.N., Spinks G.M., Wallace G.G., Mazzoldi A., Rossi D.D., Rinzler A.G., Jaschinski O., Roth S., Kertesz M. (1999). Carbon Nanotube Actuators. Science.

[21] Kim B.Y.A., Muramatsu H., Hayashi T., Endo M., Terrones M., Dresselhaus M.S. (2006). Fabrication of High Purity, Double-Walled Carbon Nanotube Buckypaper. Chem. Vap. Deposition.

[22] Park J.G., Li S., Fan X., Zhang C., Wang B. (2008). The High Current-Carrying Capacity of Various Carbon Nanotube-Based Buckypapers. Nanotechnology.

[23] Endo M., Muramatsu H., Hayashi T., Kim Y.A., Terrones M., Dresselhaus M.S. (2005). 'Buckypaper' from Coaxial Nanotubes. Nature.

[24] Park T.J., Banerjee S., Hemraj-Benny T., Wong S.S. (2006). Purification Strategies and Purity Visualization for Single-Walled Carbon Nanotubes. J. Mater. Chem..

[25] Suppiger D., Busato S., Ermanni P. (2008). Characterization of Single-Walled Carbon Nanotube Mats and their Performance as Electromechanical Actuators. Carbon.

[26] Vohrer U., Kolaric I., Haque M.H., Roth S., Detlaff-Weglikowska U. (2004). Carbon Nanotube Sheets for the Use as Artificial Muscles. Carbon.

[27] Rouse J.H. (2005). Polymer-Assisted Dispersion of Single-Walled Carbon Nanotubes in Alcohols and Applicability toward Carbon Nanotube/Sol-Gel Composite Formation. Langmuir.

[28] Bandow S., Asaka S., Zhao X., Ando Y. (1998). Purification and Magnetic Properties of Carbon Nanotubes. Appl. Phys. A: Mater. Sci. Process..

[29] Cinke M., Li J., Chen B., Cassell A., Delzeit L., Han J., Meyyappan M. (2002). Pore Structure of Raw and Purified HiPco Single-Walled Carbon Nanotubes. Chem. Phys. Lett..

[30] Dillon A.C., Gennett T., Jones K.M., Alleman J.L., Parilla P.A., Heben M.J. (1999). A Simple and Complete Purification of Single-Walled Carbon Nanotube Materials. Adv. Mater..

[31] Xu Y.Q., Peng H., Hauge R.H., Smalley R.E. (2005). Controlled Multistep Purification of Single-Walled Carbon Nanotubes. Nano Lett..

[32] Ziegler K.J., Gu Z., Peng H., Flor E.L., Hauge R.H., Smalley R.E. (2005). Controlled Oxidative Cutting of Single-Walled Carbon Nanotubes. J. Am. Chem. Soc..

[33] Hu H., Zhao B., Itkis M.E., Haddon R.C. (2003). Nitric Acid Purification of Single-Walled Carbon Nanotubes. J. Phys. Chem. B.

[34] Vaisman L., Wagner H.D., Marom G. (2006). The Role of Surfactants in Dispersion of Carbon Nanotubes. Adv. Colloid Interface Sci..

[35] Sun Z., Nicolosi V., Rickard D., Bergin S.D., Aherne D., Coleman J.N. (2008). Quantitative Evaluation of Surfactant-Stabilised Single-Walled Carbon Nanotubes:Dispersion Quality and Its Correlation with Zeta Potential. J. Phys. Chem. C.

[36] Shaffer M.S.P., Fan X., Windle A.H. (1998). Dispersion and Packing of Carbon Nanotubes. Carbon.

[37] Lin T., Bajapi V., Ji T., Dai L. (2003). Chemistry of Carbon Nanotubes. Aust. J. Chem..

[38] Yu J., Grossiord N., Koning C.E., Loos J. (2007). Controlling the Dispersion of Multi-Wall Carbon Nanotubes in Aqueous Surfactant Solution. Carbon.

[39] Wang Y., Gao L., Sun J., Liu Y., Zheng S., Kajiura H., Li Y., Noda K. (2006). An Integrated Route for Purification, Cutting and Dispersion of Single-Walled Carbon Nanotubes. Chem. Phys. Lett..

[40] Priya B.R., Byrne H.J. (2008). Investigation of Sodium Dodecyl Benzene Sulfonate Assisted Dispersion and Debundling of Single-Walled Carbon Nanotubes. J. Phys. Chem. B.

[41] Nish A., Hwang J.J., Doig J., Nicholas R.J. (2007). Highly Selective Dispersion of Single-Walled Carbon Nanotubes Using Aromatic Polymers. Nature.

[42] Zheng M., Jagota A., Semke E.D., Diner B.A., Mclean R.S., Lustig S.R., Richardson R.E., Tassis N.G. (2003). DNA-Assisted Dispersion and Separation of Carbon Nanotubes. Nature.

[43] Hou P.X., Liu C., Cheng H.-M. (2008). Purification of Carbon Nanotubes. Carbon.

[44] Hirsch A., Vostrowsky O. (2005). Functionalization of Carbon Nanotubes. Top. Curr. Chem..

[45] Dumée L., Sears K., Schütz J., Finn N., Duke M., Gray. S. (2009). Design and Characterisation of Carbon Nanotube Bucky-Paper Membranes for Membrane Distillation. Desalin. Water Treat..

[46] Hernández A., Calvo J.I., Prádanos P., Tejerina F. (1996). Pore Size Distributions in Microporous Membranes. A Critical Analysis of the Bubble Point Extended Method. J. Membr. Sci..

[47] Smajda R., Kukovecz Á., Kónya Z., Kiricsi I. (2007). Structure and Gas Permeability of Multi-Wall Carbon Nanotube Buckypapers. Carbon.

[48] Kukovecz Á., Smajda R., Kónya Z., Kiricsi I. (2007). Controlling the Pore Diameter Distribution of Multi-Wall Carbon Nanotube Buckypapers. Carbon.

[49] Whitby R.L.D., Fukuda T., Maekawa T., James S.L., Mikhalovsky S.V. (2008). Geometric Control and Tuneable Pore Size Distribution of Buckypaper and Buckydiscs. Carbon.

[50] Das R.K., Liu B., Reynolds J.R., Rinzler A.G. (2009). Engineered Macroporosity in Single-Wall Carbon Nanotube Films. Nano Lett..

[51] Casavant M.J., Walters D.A., Scmidt J.J., Smalley R.E. (2003). Neat Macroscopic Membranes of Aligned Carbon Nanotubes. J. Appl. Phys..

[52] Wang D., Song P., Liu C., Wu W., Fan S. (2008). Highly Oriented Carbon Nanotube Papers made of Aligned Carbon Nanotubes. Nanotechnology.

[53] Gonnet P., Liang Z., Choi E.S., Kadambala R.S., Zhang C., Brooks J.S., Wang B., Kramer L. (2006). Thermal Conductivity of Magnetically Aligned Carbon Nanotube Buckypapers and Nanocomposites. Curr. Appl. Phys..

[54] Hone J., Liaguno M.C., Nemes N.M., Johnson A.T., Fischer J.E., Walters D.A., Casavant M.J., Schmidt J., Smalley R.E. (2000). Electrical and Thermal Transport Properties of Magnetically Aligned Single Wall Carbon Nanotube Films. Appl. Phys. Lett..

[55] Lawson K.W., Lloyd D.R. (1997). Membrane Distillation. J. Membr. Sci..

[56] Hoang M., Bolto B., Tran T. (2009). Desalination by Capacitive Deionisation. Water.

[57] Pan L., Wang X., Zhang Y., Chen Y., Sun Z. (2009). Electrosorption of Anions with Carbon Nanotube and Nanofibre Composite Film Electrodes. Desalination.

[58] Wang X., Li M., Chen R., Huang S., Pan L., Sun Z. (2006). Electrosorption of Ions from Aqueous Solutions with Carbon Nanotubes and Nanofibers Composite Film Electrodes. Appl. Phys. Lett..

[59] Li H., Gao Y., Pan L., Zhang Y., Chen Y., Sun Z. (2008). Electrosorptive Desalination by Carbon Nanotubes and Nanofibers Electrodes and Ion-Exchange Membranes. Water Res..

[60] Dai K., Shi L., Zhang D., Fang J. (2006). NaCl Adsorption in Multi-Walled Carbon Nanotube/Active Carbon Combination Electrode. Chem. Eng. Sci..

[61] Viswanathan G., Kane D.B., Lipowicz P.J. (2004). High Efficiency Fine Particulate Filtration Using Carbon Nanotube Coatings. Adv. Mater..

[62] Kang S., Pinault M., Pfefferle L.D., Elimelech M. (2007). Single-Walled Carbon Nanotubes Exhibit Strong Antimicrobial Activity. Langmuir.

[63] Brady-Esétvez A.S., Kang S., Elimelech M.A. (2008). Single-Walled-Carbon-Nanotube Filter for Removal of Viral and Bacterial Pathogens. Small.

[64] Edwards S.L., Church J.S., Werkmeister J.A., Ramshaw J.A.M. (2009). Tubular Micro-scale Multiwalled Carbon Nanotube-based Scaffolds for Tissue Engineering. Biomaterials.

[65] Voher U., Zschoerper N.P., Koehne Y., Langowski S., Oehr C. (2007). Plasma Modification of Carbon Nanotubes and Bucky Papers. Plasma Process. Polym..

[66] Correa-Duarte M.A., Wagner N., Rojas-Chapana J., Morsczeck C., Thie M., Giersig M. (2004). Fabrication and Biocompatibility of Carbon Nanotube-based 3D Networks as Scaffolds for Cell Seeding and Growth. Nano Lett..

[67] Galvan-Garcia P., Keefer E.K., Yang F., Zhang M., Fang S., Zakhidov A.A., Baughman R.H., Romero M. (2007). Robust Cell Migration and Neuronal Growth on Pristine Carbon Nanotube Sheets and Yarns. J. Biomater. Sci., Polym. Ed..

[68] Hummer G., Rasalah J.C., Noworyta J.P. (2001). Water Conduction through the Hydrophobic Channel of a Carbon Nanotube. Nature.

[69] Skoulidas A., Ackerman D.M., Johnson K., Sholl D.S. (2002). Rapid Transport of Gases in Carbon Nanotubes. Phys. Rev. Lett..

[70] Chen H., Johnson J.K., Sholl D.S. (2006). Transport Diffusion of Gases is Rapid in Flexible Carbon Nanotubes. J. Phys. Chem. B.

[71] Waghe A., Rasaiah J.C., Hummer G. (2002). Filling and Emptying Kinetics of Carbon Nanotubes in Water. J. Chem. Phys..

[72] Noy A., Park H.G., Fornasiero F., Holt J.K., Grigoropoulos C.P., Bakajin O. (2007). Nanofluidics in Carbon Nanotubes. Nanotoday.

[73] Corry B. (2008). Designing Carbon Nanotube Membranes for Efficient Water Desalination. J. Phys. Chem. B.

[74] Thomas J.A., McGaughey J.H. (2008). Reassessing Fast Water Transport through Carbon Nanotubes. Nano Lett..

[75] Zheng J., Lennon E.M., Tsao H. K., Sheng Y. J., Jiang S. (2005). Transport of a Liquid Water and Methanol Mixture through Carbon Nanotubes under a Chemical Gradient. J. Chem. Phys..

[76] Striolo A. (2006). The Mechanism of Water Diffusion in Narrow Carbon Nanotubes. Nano Lett..

[77] Majumder S.R., Choudhury N., Ghosh S.K. (2007). Enhanced Flow in Smooth Single-File Channel. J. Chem. Phys..

[78] Allen R., Hansen J.P., Melchionna S. (2003). Molecular Dynamics Investigation of Water Permeation through Nanopores. J. Chem. Phys..

[79] Whitby M., Quirke N. (2007). Fluid flow in Carbon Nanotubes and Nanopipes. Nature.

[80] Sun L., Crooks R.M. (2000). Single Carbon Nanotube Membranes: A well-Defined Model for Studying Mass Transport through Nanoporous Materials. J. Am. Chem. Soc..

[81] Miller S.A., Martin C.R. (2002). Controlling the Rate and Direction of Electroosmotic Flow in Template-Prepared Carbon Nanotube Membranes. J. Electroanal. Chem..

[82] Miller S.A., Young V.Y., Marin C.R. (2001). Electroosmotic Flow in Template-Prepared Carbon Nanotube Membranes. J. Am. Chem. Soc..

[83] Naguib N., Ye H., Gogotsi Y., Yazicioglu A.G., Megaridis C.M., Yoshimura M. (2004). Observation of Water Confined in Nanometer Channels of Closed Carbon Nanotubes. Nano Lett..

[84] Rossi M.P., Ye H., Gogotsi Y., Babu S., Ndungu P., Bradley J.-C. (2004). Environmental Scanning Electron Microscopy Study of Water in Carbon Nanopipes. Nano Lett..

[85] Majumder M., Chopra N., Hinds B.J. (2005). Effect of Tip Functionalization on Transport through Vertically Oriented Carbon Nanotube Membranes. J. Am. Chem. Soc..

[86] Nednoor P., Chopra N., Gavalas V., Bachas L.G., Hinds B.J. (2005). Reversible Biochemcial Switching of Ionic Transport through Aligned Carbon Nanotube Membranes. Chem. Mater..

[87] Nednoor P., Gavalas V.G., Chopra N., Hinds B.J., Bachas L.G. (2007). Carbon Nanotube Based Biomimetic Membranes: Mimicking Protein Channels regulated by Phosphorylation. J. Mater. Chem..

[88] Majumder M., Zhan X., Andres R., Hinds B.J. (2007). Voltage Gated Carbon Nanotube Membranes. Langmuir.

[89] Fornasiero F., Park H.G., Holt J.K., Stadermann M., Grigoropoulos C.P., Noy A., Bakajin O. (2008). Ion Exclusion by Sub-2-nm Carbon Nanotube Pores. Proc. Natl. Acad. Sci. USA.

[90] Holt J.K., Noy A., Huser T., Eaglesham D., Bakajin O. (2004). Fabrication of a Carbon Nanotube-Embedded Silicon Nitride Membrane for Studies of Nanometer-Scale Mass Transport. Nano Lett..

[91] Sears K., Schütz J., Huynh C., Hawkins S., Humphries W. Evaluation and characterisation of carbon nanotube membranes. Proceedings of the 2008 International Conference On Nanoscience and Nanotechnology (ICONN).

[92] Murakami Y., Chiashi S., Einarsson E., Maruyaman S. (2005). Polarization Dependence of Resonant Raman Scattering from Vertically Aligned Single-Walled Carbon Nanotube Films. Phys. Rev. B: Condens. Matter Mater. Phys..

[93] Duesberg G.S., Loa I., Burghard M., Syassen K., Roth S. (2000). Polarized Raman Spectroscopy on Isolated Single-Wall Carbon Nanotubes. Phys. Rev. Lett..

[94] Gommans H.H., Alldredge J.W., Tashiro H., Park J., Magnuson J. (2000). Fibers of Aligned Single-Walled Carbon Nanoutbes: Polarized Raman Spectroscopy. J. Appl. Phys..

[95] Yu M., Funke H.H., Falconer J.L., Noble R.D. (2009). High Density, Vertically-Aligned Carbon Nanotube Membranes. Nano Lett..

[96] Futaba D.N., Hata K., Yamada T., Hiraoka T., Hayamizu Y., Kakudate Y., Tanaike O., Hatori H., Yumura M., Iijima S. (2006). Shape-Engineerable and Highly Densely Packed Single-Walled Carbon Nanotubes and Their Application as Super-Capacitor Electrodes. Nature.

[97] Chakrapani N., Wei B., Carrillo A., Ajayan P.M., Kane R.S. (2004). Capillarity-Driven Assembly of Two-Dimensional Cellular Carbon Nanotube Foams. Proc. Natl. Acad. Sci. USA.

[98] Jung H.Y., Jung S.M., Gu G.H., Suh J.S. (2006). Anodic Aluminum Oxide Membrane Bonded on a Silicon Wafer for Carbon Nanotube Field Emitter Arrays. Appl. Phys. Lett..

[99] Velleman L., Shapter J.G., Losic D. (2009). Gold Nanotube Membranes Functionalised with Fluorinated Thiols for Selective Molecular Transport. J. Membr. Sci..

