# Supplementary material for: Recent Developments in Carbon Nanotube Membranes for Water Purification and Gas Separation
Source: Materials (Basel). 2010 Jan 4;3(1):127–49. doi: 10.3390/ma3010127 (PMC5510185; doi:10.3390/ma3010127)
Supplement: Supplementary file 1 [file materials-03-00127-s001.zip › Supplementary Materials-Tables.pdf]

Supplementary Materials

Recent Developments in Carbon Nanotube Membranes for Water Purification and Gas Separation

Kallista Sears [1,*], Ludovic Dumée [1,2], Jürg Schütz [1], Mary She [3], Chi Huynh [1], Stephen Hawkins [1], Mikel Duke [2] and Stephen Gray [2]

S1. Experimental Details

S1.1. CNT growth

Two types of CNTs, referred to as fine and coarse, were grown for our membrane work via different CVD processes. The process used to grow the fine CNTs involves depositing an iron cataylst film (~5 nm) onto a Si substrate bearing a thin (100–600 nm) SiO_2 layer. Acetylene (5%) in Helium is used as the carbon feedstock and heated to between 650 and 750 °C [1]. This process produces CNTs which are largely free from structural blockages and are therefore ideal for the isoporous membrane work. In the case of the Bucky-paper membranes, both fine and coarse CNTs were used. The coarse CNTs were produced by continuously injecting a ferrocene/acetylene mixture at a temperature of ~700 °C. This mixture provides both the carbon feedstock and iron catalyst for CNT growth. Substrates of quartz or silicon (with a thin SiO_2 layer) were used with no iron catalyst film.

S1.2. Isoporous CNT Membrane Construction

Forests of “fine” CNTs (see Table 1) were infiltrated with a two part epoxy resin system. The infiltrated CNT forest is then cured overnight at 120°C. The excess epoxy and silicon substrate were removed by polishing with progressively finer diamond grit followed by a 2–4 hour, high frequency plasma treatment with 30% O_2 in Ar, at a pressure of 0.6 mbar and power of 80 W in a Pico PC system from Diener Electronics (http://www.plasma-etcher.com/36-0-pico.html).

S1.3. Bucky-Paper Membrane Construction

Bucky-paper membranes were prepared by dispersing CNTs (either fine or coarse) into analytical grade isopropanol by repeated sonication (15 minute intervals at 150 W) and stirring. Once a well dispersed solution was achieved it was immediately filtered through a poly(ether-sulfone) (PES) support of 0.22 µm pore size using a 47 mm diameter Millipore filtration unit and house line vacuum (ΔP= -95 kPa). Please refer to references [2,3] for further details on Bucky-paper membrane construction.

S1.4. Membrane Characterisation Techniques

Scanning electron microscopy (SEM) was performed using a Philips SEM FEG at 2 kV and a working distance of ~9 mm. The as grown CNT forests and ones that were epoxy infiltrated were coated with a thin iridium layer to minimize charging effects. However the CNT Bucky-paper samples were sufficiently conductive to not require coating.

An FEI Nova Nanolab 200 Dual Beam Focused Ion Beam (FIB) was used to (i) form cross sections of the Bucky-paper membranes and (ii) prepare TEM samples of the epoxy infiltrated CNT forests. Cross-sections of the Bucky-paper were milled with a 1 nA, 30 kV Ga ion beam, followed by 0.3 nA cleaning steps. SEM imaging of the milled cross-sections was performed at a working distance of 5 mm, 5 kV beam and a sample tilt of 52° (due to the FIB configuration).

Raman spectra were measured with a Renishaw RM 2000 confocal micro-Raman system in a backscattering configuration using a 782 nm laser at a power of ~1.43 mW. This laser power corresponds to a power density of ~2×10^4 W/cm^2 which avoided modification or damage to the CNTs. This power also minimized fluorescence from the epoxy matrix used in the isoporous CNT membranes. Raman spectra taken of the epoxy alone revealed a number of peaks including a dominant one at 1,450 cm^{-1}. However, under the above mentioned conditions, the 1,450 cm^{-1} peak was absent or negligible for the infiltrated CNT forest samples, indicating that epoxy fluorescence is not an issue. The system was calibrated with reference to the silicon 521 cm^{-1} and diamond 1,332 cm^{-1} peaks. A Bucky-paper sample, in which the CNTs are randomly orientated, was used as a control. As expected, it exhibited no change in Raman intensity with changing polarisation of the incident laser.

Please refer to references [2,3] for further details regarding: particle exclusion tests, BET surface area measurements, contact angle measurements, and the membrane distillation setup.

S1.5. Gas Permeance

Gas permeance measurements were performed by placing the membrane in an o-ring sealed holder which separates a large upstream (feed) vessel from a much smaller downstream (permeate) vessel. To ensure integrity of the o-ring seal, leak rate checks were performed with control membranes that were gas impermeable. After loading the membrane, both the feed and permeate vessels were evacuated. The feed vessel was then isolated from both the vacuum and membrane holder, and filled to atmospheric pressure with filtered, dehumidified air. Prior to testing, the membrane was conditioned for 1 hour by maintaining vacuum on the permeate side while opening the membrane to the feed vessel. For testing, the permeate side was isolated from vacuum and the pressure rise monitored over time until equilibrium was reached. The feed pressure remains essentially constant due to its much larger volume compared to that of the permeate. The membrane permeance can then be determined by fitting the pressure rise, $P_P(t)$, in the permeate vessel with the following equation:

$$P_P(t) = P_F\left(1 - \frac{P_F - P_o}{P_F} e^{\left(\frac{-RTfA}{V}\right)t}\right)$$

Where P_F is the constant pressure in the feed vessel, P_o is the initial pressure of the permeate vessel, R is the universal gas constant, V is the volume of the permeate vessel, T is the temperature, A is the exposed membrane area, and f [moles m^{-2}s^{-1}Pa^{-1}] is membrane permeance.

Table S1. Summary of N_2 BET surface area results reported in the literature for carbon nanotubes.

Group	CNT Type	Synthesis	Treatments	Walls	Outer Diameter (nm)	Surface Area (g/m^2)	BP†
Onyesták 2003[4]	MWNT	CVD	None (<87.5 wt % CNT)	7	9.4 ± 3	130	No
			purified	7		250	
Smajda 2007[5]	MWNT	CVD	purified (95 wt % CNT)	22	15 - 25	197.7	Yes
Muramatsu 2005[6]	DWNT	CVD	purified (95 wt % CNT)	2	1.41,1.56	569	Yes
	SWNT	HiPco (Carbon Nano-technology)	Used as purchased (but highly purified)	1	0.85 - 1.26	642	Yes
Cinke et al. 2002[7]	SWNT	HiPco	none (22 wt % Fe)	1	0.93 - 1.35	577	No
			purified (< 0.4 wt % Fe)*	1		1587	
Inoue 1998[8]	MWNT	Hyperion Catalysis Int. Co.	no further purification	9	10	268	No
Cooper 2003[9]	SWNT	Laser ablation (Johnson Space Center)	purified (10.5 wt % impurity)	1	Not specified	350–450	BP
Eswaramoorthy 1999[10]	SWNT	Arc discharge	None	1	1.1	376	No
			HCl	1	1.1	483	No
			HNO_3	1	1.1	429	No
Yang 2002[11,12]	SWNT	HiPco (Carbon Nano-technology)	no further purification (27 wt % Fe)	1	0.8–1.2	524	No
			HCl (18 wt % Fe)	1	0.8–1.2	587	No
			oxidation+HCl (6 wt % Fe)	1	0.8–1.2	861	No
CSIRO	MWNT	CVD	none (>95 wt % CNT)	6	9	197	BP
	MWNT	CVD	none (>90 wt % CNT)	37	37	36	BP

* Purification included an initial step to debundle the CNTs.

† This column indicates whether the measurements were made on a CNT Bucky-paper (BP).

Table S2. Summary of isoporous CNT membrane properties, permeance and permeabilities taken from the literature.

Group	Matrix Material	Treatments	Thick-ness (μm)	Diameter (nm)	CNT Density (10^9 cm^{-2})	Test gas	Permeability (10^{-13} mol $m^{-1}s^{-1}Pa^{-1}$)	Permeance (10^{-8} mol $m^{-2}s^{-1}Pa^{-1}$) Measured	Knudsen[b]	Factor	Knudsen Number
Mi[13]	Polystyrene	polish + HNO_3	10	6.3	1.87	N_2	3.2	3.2	1.9	1.7	10.3
Kim [14]	Polysulfone	amine –functionalized CNTs	0.6	1.6	70	He	6	100	64	1.6[c]	81.3
Hinds[15,16]	Polystyrene	PS+ H2O	5	7.5	60	N_2	100	260	233	1.1	8.7
Holt [17]	Si_3N_4	RIE	2.6	1.6	250	air	283	1170	19.6	59.7	40.6
	PC[a] control (15 nm)	N/A	6	15	0.6	air	29.5	49	16.8	2.1	4.3
Yu [18]	Densified forest only	Water etching	750	3.6±0.9	2900	N_2	751000	10000	8.98	1114.8	18.1
CSIRO	epoxy	None	35	4.5	50	air	0.05	0.015	6.5	-	14.4
	epoxy	polish + O_2/Ar plasma	35	4.5	50[d]	air	0.3	0.1	6.5	-	14.4
	PC[a] control (10 nm)	N/A	6	10	0.6	air	32.5	53.5	5.0	10.7	6.5
	PC[a] control (30 nm)	N/A	6	30	0.6	air	380	630	134	4.7	2.2

[a] PC = a polycarbonate track etched membrane.

[b] The theoretical permeance was calculated using Knusdsen diffusion. A tortuosity factor of 1 was assumed except in the case of Hinds and Mi, where tortuosity values of 1.1 and 1.26 were used, respectively, as reported in their publications.

[c] Kim *et al* report an enhancement factor ~2 times larger than that calculated by us.

[d] Assuming that all the as grown CNTs are contributing to permeance.

References

1. Huynh, C.; Hawkins, S. Understanding the Synthesis of Directly Spinnable Carbon Nanotube Forests. *Carbon* **2009**, submitted.
2. Dumée, L.F.; Sears, K.; Schütz, J.; Finn, N.; Huynh, C.; Hawkins, S.; Duke, M.; Gray, S. Characterisation and Evaluation of Carbon Nanotube Bucky-paper Membranes for Direct Contact Membrane Distillation . *J. Membr. Sci.* **2009**, submitted,
3. Dumée, L.; Sears, K.; Schütz, J.; Finn, N.; Duke, M.; Gray., S. Design and Characterisation of Carbon Nanotube Bucky-Paper Membranes for Membrane Distillation. *Desalin. Water Treat.* **2009**, in press.
4. Onyestyák, Gy.; Valyon, J.; Hernádi, K.; Kiricsi, I.; Rees, L.V.C. Equilibrium and Dynamics of Acetylene Sorption in Multiwalled Carbon Nanotubes. *Carbon* **2003**, *41*, 1241–1248
5. Smajda, R.; Kukovecz, Á.; Kónya, Z.; Kiricsi, I. Structure and Gas Permeability of Multi-Wall Carbon Nanotube Buckypapers. *Carbon* **2007**, *45*, 1176–1184
6. Muramatsu, H.; Hayashi, T.; Kim, Y.A.; Shimamoto, D.; Kim, Y.J.; Tantrakarn, K.; Endo, M.; Terrones, M.; Dresselhaus, M.S. Pore Structure and Oxidation Stability of Double-Walled Carbon Nanotube-Derived Bucky Paper. *Chem. Phys. Lett.* **2005**, *414*, 444–448
7. Cinke, M.; Li, J.; Chen, B.; Cassell, A.; Delzeit, L.; Han, J.; Meyyappan, M. Pore Structure of Raw and Purified HiPco Single-Walled Carbon Nanotubes. *Chem. Phys. Lett.* **2002**, *365*, 69–74
8. Inoue, S.; Ichikuni, N.; Suzuki, T.; Uematsu, T.; Kaneko, K. Capillary Condensation of N_2 on Multiwall Carbon Nanotubes. *J. Phys. Chem. B* **1998**, *102*, 4689–4692.
9. Cooper, S.M.; Chuang, H.F.; Cinke, M.; Cruden, B.A.; Meyyappan, M. Gas Permeability of a Buckypaper Membrane. *Nano Lett.* **2003**, *3*, 189–192
10. Eswaramoorthy, M.; Sen, R.; Rao, C.N.R. A Study of Micropores in Single-Walled Carbon Nanotubes by the Adsorption of Gases and Vapors. *Chem. Phys. Lett.* **1999**, *304*, 207–210.
11. Yang, C.-M.; Kaneko, K.; Yudasaka, M.; Iijima, S. Effect of Purification on Pore Structure of HiPco Single-Walled Carbon Nanotube Aggregates. *Nano Lett.* **2002**, *2*, 385–388.
12. Yang, C.-M.; Kim, D.Y.; Lee, Y.H. Formation of Densely Packed Single-Walled Carbon Nanotube Assembly. *Chem. Mater.* **2005**, *17*, 6422–6429.
13. Mi, W.; Lin, Y.S.; Li, Y. Vertically Aligned Carbon Nanotube Membranes on Macroporous Alumina Supports. *J. Membr. Sci.* **2007**, *304*, 1–7.
14. Kim, S.; Jinschek, J.R.; Chen, H.; Sholl, D.S.; Marand, E. Scalable Fabrication of Carbon Nanotube/Polymer Nanocomposite Membranes for High Flux Gas Transport. *Nano Lett.* **2007**, *7*, 2806–2811.
15. Hinds, B.J.; Chopra, N.; Rantell, T.; Andrews, R.; Gavalas, V.; Bachas, L.G. Aligned Multiwalled Carbon Nanotube Membranes. *Science* **2004**, *303*, 62–65.
16. Majumder, M.; Chopra, N.; Andrews, R.; Hinds, B.J. Enhanced Flow in Carbon Nanotubes. *Nature* **2005**, *438*, 44.
17. Holt, J.K.; Park, H.G.; Wang, Y.; Staderman, M.; Artyukhin, A.B.; Grigoropoulos, C.P.; Noy, A.; Bakajin, O. Fast Mass Transport Through Sub-2-Nanometer Carbon Nanotubes. *Science* **2006**, *312*, 1034–1037.

18. Yu, M.; Funke, H.H.; Falconer, J.L.; Noble, R.D. High density, Vertically-Aligned Carbon Nanotube Membranes. *Nano Lett.* **2009**, *9*, 225–229.

© 2010 by the authors; licensee Molecular Diversity Preservation International, Basel, Switzerland. This article is an open-access article distributed under the terms and conditions of the Creative Commons Attribution license (http://creativecommons.org/licenses/by/3.0/).
